# The cell organization underlying structural colour is involved in *Flavobacterium* IR1 predation

**DOI:** 10.1038/s41396-020-00760-6

**Published:** 2020-09-01

**Authors:** Raditijo Hamidjaja, Jérémie Capoulade, Laura Catón, Colin J. Ingham

**Affiliations:** 1 Hoekmine BV, Utrecht, 3548 CH The Netherlands; 2 Department of Bionanoscience, Kavli Institute of Nanoscience, Delft University of Technology, van der Maasweg 9, Delft, 2629 HZ The Netherlands; 3 Department of Chemistry, Cambridge University, Lensfield Road, Cambridge, UK

**Keywords:** Water microbiology, Microbial ecology

## Abstract

*Flavobacterium* IR1 is a gliding bacterium with a high degree of colonial organization as a 2D photonic crystal, resulting in vivid structural coloration when illuminated. *Enterobacter cloacae* B12, an unrelated bacterium, was isolated from the brown macroalga *Fucus vesiculosus* from the same location as IR1. IR1 was found to be a predator of B12. A process of surrounding, infiltration, undercutting and killing of B12 supported improved growth of IR1. A combination of motility and capillarity facilitated the engulfment of B12 colonies by IR1. Predation was independent of illumination. Mutants of IR1 that formed photonic crystals less effectively than the wild type were reduced in predation. Conversely, formation of a photonic crystal was not advantageous in resisting predation by *Rhodococcus* spp. PIR4. These observations suggest that the organization required to create structural colour has a biological function (facilitating predation) but one that is not directly related to the photonic properties of the colony. This work is the first experimental evidence supporting a role for this widespread type of cell organization in the *Flavobacteriia*.

## Introduction

*Flavobacterium* IR1 is a gliding bacterium that can spread over hydrated surfaces [1]. Gliding and spreading by IR1 appears to use many of the Spr and Gld proteins homologous to those from *Flavobacterium johnsoniae* [1–4]. Metabolism of the sulphated polysaccharides κ-carageenan and fucoidan by IR1 suggests interactions between this bacterium and both red and brown macroalgae. Like some other members of the order *Flavobacteriia* [5–7] colonies of IR1 cells are intensely coloured when illuminated with white light and viewed from specific angles [1]. This is due to highly repeated arrays of cells which can assemble rapidly to form a 2D photonic crystal (2DPC) [1], a form of structural colour (SC), which is distinct in mechanism from chemical pigmentation. SC, in various forms, is widely distributed in nature with roles in sexual selection, camouflage, modulation of photosynthesis and thermal regulation [8–10]. However, no role for bacterial SC has been identified to date.

Surfaces form one of the major habitats of microbial life [11, 12]. Bacteria compete on surfaces with diverse strategies. Swarming, gliding and other mechanisms of bacterial spreading over surfaces offer advantages in dispersal and competition [12–15]. Predators, including bacteria, generally require motility to contact their prey. The gliding myxobacteria actively sense, surround and consume other bacteria [16–18]. Flavobacteria glide by a mechanism distinct from surface motility in myxobacteria [2, 19]. This form of gliding is rapid and often associated with the enzymatic degradation of polysaccharide as well as improved survival on surfaces [12]. Motile, bacterial predators that target both *Eukaryotes* and other bacteria are widely distributed in nature [20–22]. It has been suggested that the predator–prey relationship originated within the *Bacteria* [23] and that bacterial predation may play a significant ecological role [12]. Within the gliding *Flavobacteriia* predators of *Eukaryotes* have been described, such as a ‘*Cytophaga’* sp. consuming diatoms [24], and bacteriolytic *Olleya* and *Tenacibaculum* species [25]. The soil bacterium *F. johnsoniae* is predatory, deploying a type VI secretory apparatus that delivers toxins to Gram-negative bacterial prey [26].

IR1 has not previously been shown to be predatory on other bacteria. This work focuses on interactions with bacteria isolated from the same environment as IR1. We show that predation by IR1 of other bacteria occurs, and requires organization as a 2DPC. This is the first experimental evidence that formation of a photonic crystal by bacteria has survival value, albeit not one directly linked to illumination.

## Results

### IR1 invades colonies of other bacteria on low-nutrient agar plates

A screening was made for bacteria that interacted when in close proximity with IR1 colonies on ASWBLow agar plates. The source of the bacteria was the same as IR1: sediment and the brown alga *Fucus vesiculosus* from brackish water near Rotterdam Harbour (NL), after storage of original samples at −80 °C for 5 years (Tables S1 and S2 for strains used). The most common form of interaction found was that motile, gliding cells from colonies of IR1 overgrew and degraded some adjacent colonies. The bacteria that were vulnerable to IR1 were identified on the basis of 16S rRNA sequencing and found to be *Moraxella osloensis*, *Staphylococcus pasteuri, Pseudomonas spp., Pedobacter spp*. and *Enterobacteria cloacae*. The latter were repeatedly isolated and strain B12 was chosen for further work. In contrast, successful competition by IR1 over B12 was not seen in liquid culture or a submerged biofilm model (Supplementary Fig. S1). SC was also not observed in liquid culture nor biofilms.

### Competition between IR1 and B12 co-inoculated on an agar surface

Further competition experiments were performed between IR1 and GFP-expressing B12(pGFP) on low-nutrient agar plates to determine the basis of the competitiveness of IR1. The two strains were co-inoculated as a 10 µl spot on ASWBLow plates, which were then incubated at 22 °C for up to 2 days. Within the area of inoculation, IR1 reduced the numbers of viable B12 to below the initial inoculation level, suggesting an active killing mechanism. Replacing the cells of B12 with similar numbers of fluorescein-labelled latex spheres (0.2–2 µm diameter) resulted in no significant redistribution of the spheres by growing IR1. This suggests that IR1 was not simply pushing bacterial-sized objects outside the imaging area. Outside the area of (co-)inoculation, the more motile IR1 dominated completely (Fig. S2b, c) and was able to disengage from B12 and form axenic gliding groups. Imaging of B12(pGFP) and IR1 indicated that B12 was not present within emerging masses of IR1 (Fig. S2b, c).

### IR1 grows on living cells of B12 on starvation medium suggesting predation

**Fig. 1:**
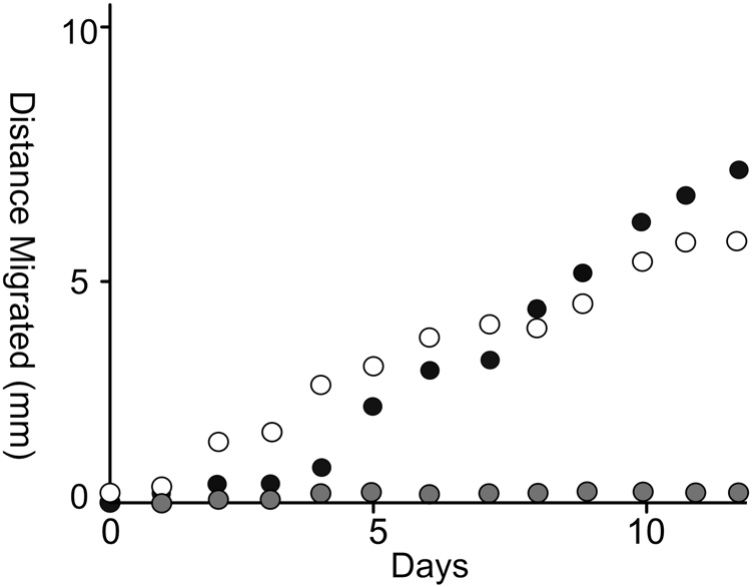
Colony expansion of IR1 on starvation medium in the presence or absence of B12. IR1 colony expansion rates (average of *n* = 3) were calculated over two weeks. Shaded circles, IR1 on agar without B12. Solid circles, IR1 on agar covered with living B12. Open circles, IR1 on agar with dead B12.

The interaction between IR1 and B12 was tested on agar plates that contained insufficient nutrients for the growth of either strain alone (starvation medium). IR1 was inoculated directly on a starvation plate previously spread with either dead or alive B12 (that had been repeatedly washed to avoid carry-over of nutrients). Both dead and living B12 cells supported progressive colony expansion (up to 0.5 mm day^−1^) by IR1 over a period of 12 days, compared to starvation medium alone (Fig. 1 and Fig. S2d). Live strain B12, in the absence of IR1, did not show a high level of propidium iodide (PI) staining on starvation medium and ASWLow suggesting that autolysis was not occurring (Fig. S3a, b). Therefore, IR1 appeared to be growing at the expense of B12; that scavenging (use of dead cells as nutrients) and predation (use of live cells of another species as nutrients) both occurred.

### Invasion of B12 by IR1 is first by infiltration and then by undercutting of B12

In order to visualize the early stages in predation, an assay was created where spots of IR1 and B12(pGFP) were inoculated 3 mm apart on ASWBLow agar. This “encounter” assay allowed growth of both strains, motility of IR1 but not B12, and monitoring by microscopy of the early interactions upon contact. Initially, IR1 expanded equally in all directions, showing no directed movement towards the B12 colony. Contact between two colonies (on the mm scale) was therefore driven by gliding IR1 and was accidental, not directed. After contact, the following stages in predation were observed:

#### Stage 1 (1–4 h after contact)

**Fig. 2:**
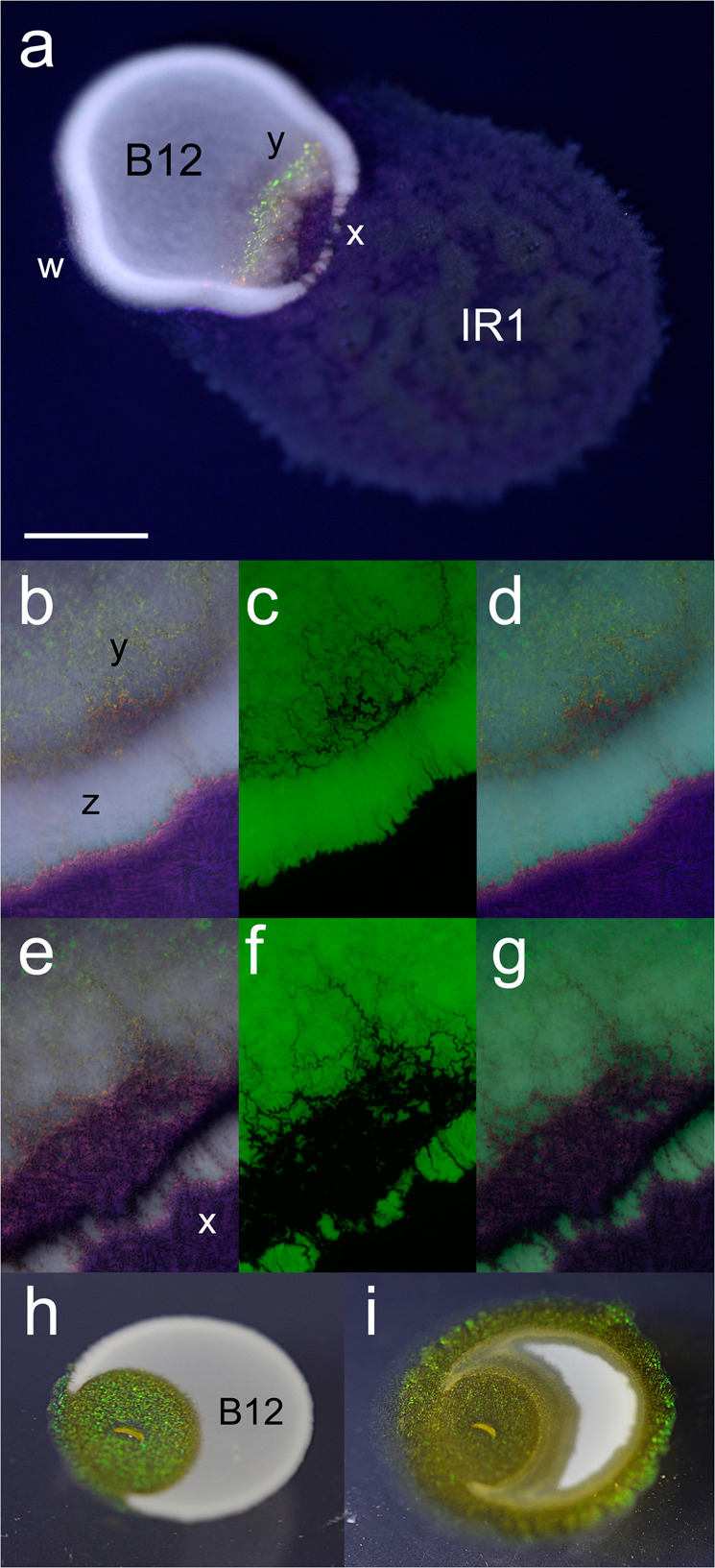
IR1 invades and predates adjacent colonies of B12. **a** Inoculation of IR1 adjacent to B12(pGFP) on ASWBFLow plates (ASWBLow agar supplemented with 0.5% w/v fucoidan), showing the result 10 h after contact between the spreading colony of IR1 and the static mass of B12. IR1 surrounds the B12 colony (w) and creates breaches (x) in the thicker edge of the B12 colony and a shift from dull purple/red SC typical of growth on ASWBFLow to green (y). IR1, IR1 colony; B12, B12 colony. **b**–**d** Images 4 h after contact with invading IR1. **b** Illumination from side showing white B12, with a thicker colony at the periphery (z) and SC from IR1 (bright pinpoints of colour including deep within the B12 colony) (y). **c** Fluorescence image showing GFP expressed by B12. **d** Merged (**b**) and (**c**). **e**–**g** are similar to **b**–**d** but after 9 h showing more extensive clearing of B12 cells and major breaches at periphery of the B12 colony (x). **h** and **i** show an experiment where B12 is inoculated in a droplet on to starvation medium, allowed to dry and then IR1 inoculated inside B12. **h** Result after 4 days showing expansion of the IR1 colony (IR1, showing predominantly green SC) to breach the periphery of the B12 colony (opaque white) from within. **i** Result of the same colony as (**h**) after 8 days showing progressive destruction of the B12 colony and movement around the periphery of B12 to engulf it. Scale bar indicates 0.4 mm for (**a**), 0.15 mm for (**b**–**g**) and 0.5 mm for (**h**) and (**i**).

Cells of IR1 infiltrated the B12 colony. The IR1 cells were flexible (Movie S1) and moved through dense masses of B12. In addition, IR1 cells moved around the periphery of the B12 colony to surround it, as detectable by the SC displayed by IR1 (Fig. 2a).

#### Stage 2 (4–20 h after contact)

Channels were created through the periphery of the B12 colony by groups of IR1 (Fig. 2a–d).

#### Stage 3 (after 20 h)

**Fig. 3:**
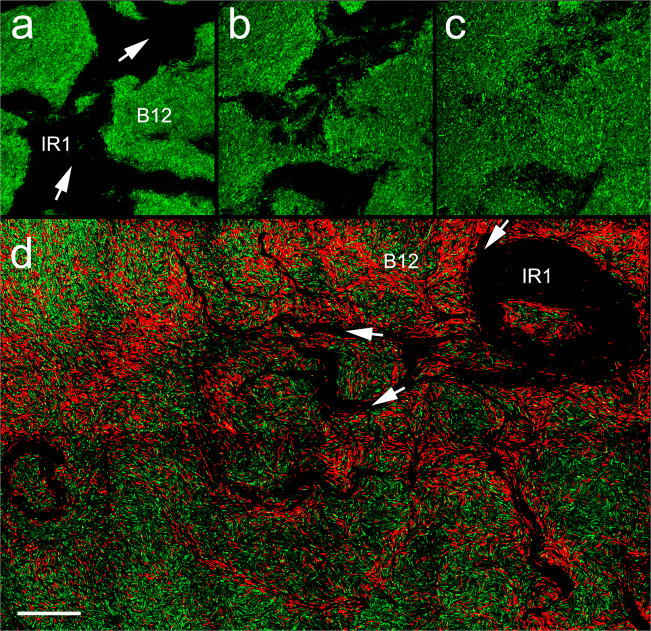
Invasion of B12 by IR1 imaged by confocal microscopy. **a**–**c** Three images taken from a Z-slice of a colony of B12(pGFP) during predation by IR1 (unstained, lines of advance shown with white arrows). From left to right the three slices show B12 cells at the agar surface, then 5, and 10 µm heights. **d** Overview image assembled from multiple contiguous images showing IR1 penetrating a colony of B12(pGFP). IR1 (not stained, visible as dark root-like regions but with an overall invasion route of top right to bottom left) is moving into a colony of GFP-expressing B12. White arrows show the direction of movement of some of the IR1 masses. Propidium iodide (red) is staining damaged cells (predominantly B12) within 20 μm of the major lines of advance of IR1. The scale bar in (**d**) indicates 50 µm when applied to (**a**–**c**) and 80 µm when applied to (**d**).

Penetration of IR1 cells into the B12 colony interior occurred through increasingly large breaches at the periphery of the prey colony, spreading to hollow it out. In this stage, groups of hundreds to thousands of cells of IR1 moved into B12, in an arrangement reminiscent of roots pushing through soil (Figs. 2e–g, 3 and Movie S2). Initial progress through the B12 colony was rapid, up to 60% of the rate at which IR1 spread over agar in the absence of B12, i.e., up to 5 mm h^−1^.

Because of the intense SC displayed, shifts in the organization of IR1 cells could be inferred from alterations in colour visible during invasion of the B12 colony. When the agar medium contained high levels of fucoidan, the predominant colour displayed by IR1 was a dull red purple/red (Fig. 2a, b). However, SC was more noticeable when IR1 contacted B12 and particularly an intense green colour within the B12 colony. This suggested a high degree of local organization, as a 2DPC [1], when IR1 was interacting with B12. It was notable that both the steps in predation described above, and formation of the 2DPC, were unaffected by illumination (using a broad-spectrum white LED which was optimal for viewing SC) over a 48 h period.

Inoculation of IR1 inside a larger spot of B12 on starvation medium resulted in the growth of both strains (particularly IR1); IR1 both formed a uniform SC and degraded the B12 until it reached the edge of the colony (Fig. 2h, i). At this point, IR1 then rapidly moved around the periphery of the B12 colony in less than a day, effectively engulfing it (Fig. 2h, i).

Confocal microscopy of B12(pGFP) at leading edges of IR1 during stages 2 and 3, at different depths, indicated that groups of cells of the invading IR1 were able to undercut B12 (Fig. 3a–c); i.e., the front edge of IR1 made the greatest progress into dense masses of B12 at the agar surface. IR1 interposed a dense mass of cells between the nutrient-containing surface and the mass of B12 cells above. However, after that point (50 µm behind the leading edge) IR1 cells extended from bottom to top of the colony, i.e., over 20 µm in height. This was the case for a high-density colony (inoculation of at least 5 × 10^8^ cells cm^−2^) of B12.

### The killing of B12 by IR1 is short range and inhibited by excess nutrients

On rich medium, i.e., ASWBC or ASWB agar (both containing 5 g l^−1^ peptone, the former containing 5 g l^−1^ κ-carageenan in addition to the other components of ASWBLow agar), IR1 was motile but failed to predate B12 during the first 4 days of contact. On ASWBLow plates, during invasion of a B12 colony confocal microscopy of B12(pGFP) cells immediately adjacent to the invading IR1 did not reveal any change in morphology of B12 (Movie S2 and Fig. S3). In order to investigate the action of IR1 on B12, predation assays were created in which B12(pGFP) and IR1 were inoculated adjacently as before, but PI was used to stain damaged cells [27]. Imaging by confocal microscopy suggested that the cells of B12(pGFP) were absent from the main invading groups of IR1. The cells of B12 in close proximity to the leading masses of IR1 (<30 µm) were strongly stained with PI, suggesting cells of B12 were damaged or killed by IR1 (Fig. 3d). In order to verify that cell death was primarily from B12, and not IR1, a series of co-inoculation assays were performed, both using IR1 expressing GFP inoculated onto a lawn of B12 without GFP; and the converse with a lawn of B12 (GFP) inoculated with non-fluorescent IR1. Recovery of cells, PI staining and visualization and quantification by fluorescence microscopy indicated that cell death was predominantly from B12 and not IR1 after 30 h incubation (Fig. S3c–f).

Filtered extracts from predated colonies failed to induce PI staining of B12. To further test the proposition that B12 killing was close range, rather than due to a diffusible antimicrobial molecule, an experiment was devised where IR1 and B12 were separated by a thin (60 µm), highly porous ceramic sterile filter that could not be penetrated by either species but which was permeable to even large enzyme complexes (Fig. S4). Despite incubation for up to 4 days, the ceramic membrane protected the B12 colony from IR1, as assessed by fluorescence microscopy. Therefore, the killing of B12 during predation was likely to be contact mediated.

### Surrounding of prey colonies is due to a combination of capillarity and gliding motility

As described above, when a spreading IR1 colony contacted a mass of B12 it was notable that, after 36 h, the distance spread was twofold to threefold further (tracing a minimal path around the periphery of the B12 colony) than for the leading edge of the part of the colony heading directly away from the prey bacteria. In order to test the cause of this, a series of dummy colonies were created out of plastic and wax; the results were similar to those for a real B12 colony—both objects were engulfed. On ASWBLow agar the volume of the colony appeared constant, whether or not a colony-like object was surrounded, suggesting that in this scenario the dead area of the target colony was displacing IR1, with the gliding bacterium flowing around the edge of objects. When the experiments were repeated using ASWBLow agar containing tetracycline or clindamycin (both at 10 µg ml^−1^) to prevent protein synthesis by IR1, IR1 was still able to surround colony-shaped objects, suggesting that neither growth nor new protein synthesis was required to engulf prey.

**Fig. 4:**
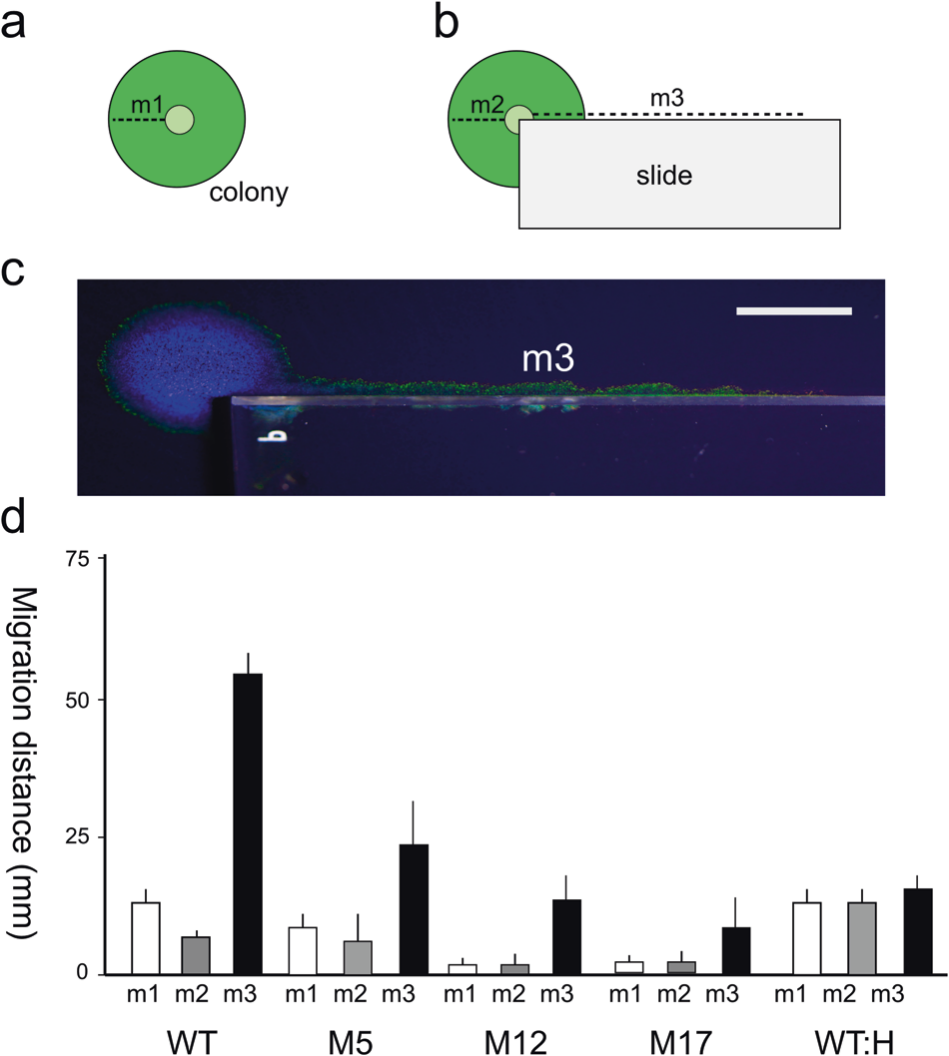
Edge effects on colony dispersal for both WT and mutants of IR1. **a**, **b** Illustration of experimental set up. **a** Simple colony on agar with the increase in radius measured after 36 h (measurement of migration distance expressed as the colony radius, m1). **b** Same inoculation method but with a sterile microscope slide positioned as shown; m2 is the colony radius as for m1; m3 is migration distance along long axis of the slide. **c** Photograph of movement from a colony of IR1 along the long axis of a glass slide (m3). Scale bar indicates 1 cm. **d** Quantification of m1, m2 and m3 for the WT and mutants M5, M12, and M17 (average of 3 experiments, ±SD) and for the WT strain using a hydrophobic microscope slide (WT:H).

When a colony of IR1 had the dual possibilities of spreading outwards over agar or along a linear edge provided by a glass slide, both events occurred but spreading along the slide was faster (Fig. 4). In this experiment there was a small decrease in the distance migrated over agar (5 mm day^−1^, compared to 8 mm day^−1^ for the same inoculum on agar without the barrier). This suggests that sufficient bacteria were leaving the colony via the edge of the glass slide to affect the expansion rate of the cells traversing open agar. When the motility deficient, non-spreading IR1 mutants M12 or M17 were used in place of the wild type (WT), a reduced rate of advancement along the slide was observed. Mutant M5, as motile as IR1 on rich medium (but a slightly less effective spreader on ASWBLow agar), showed a similar trend, with the edge of the slide facilitating a more effective spread than the motility deficient mutants but less than the WT. These data suggested that dispersal was partly an active process driven by gliding but some passive spreading was also involved. When the glass slide was siliconized to make it hydrophobic, a meniscus failed to form between the edge of the cover slip and the agar and so drops of nigrosine dye failed to spread along the edge. Rapid dispersal of IR1 (both motile WT and motility deficient mutant M17) was not facilitated by this hydrophobic edge (Fig. 4). These experiments indicated that some types of topography actively enhanced dispersal of a gliding bacterium when driven by gliding motility. Taken together, these data suggest that motile IR1 responds to edges and that this facility gives a significant advantage in capturing territory at the expense of less motile microorganisms and is the agency through which the colonies of other bacteria are surrounded, thus enhancing predation.

### The predation range of IR1 is broad

Predation assays were repeated for other bacteria and fungi. IR1 degraded both Gram-positive and Gram-negative bacteria (Table S2). However, the capsulated Gram-negative bacterium *Klebsiella pneumoniae* and the swarming bacterium *Proteus mirabilis* were refractory to IR1. Predation of *Candida albicans*, a yeast, also occurred. We note that IR1 approached all strains tested (on a mm scale) at a similar rate irrespective of whether predation was possible. These observations suggest that there is no specific sensing of prey bacteria before contact, and the “predator taxis” that has been observed in myxobacteria [17] is not a feature of IR1. *F. johnsoniae* has also been reported to be a predator of Gram-negative bacteria; this relative of IR1 can also predate B12 and other microorganisms, apparently in a similar way to IR1, but only under low-salt conditions (Table S3 and Fig. S5a).

### IR1 surrounds but does not infiltrate or predate colonies of other *Flavobacteriia*

IR1 was inoculated adjacent to *F. johnsoniae* UW101 to assess its interaction with other flavobacteria. IR1 surrounded the other strain when the IR1 colony expanded sufficiently faster than the *F. johnsoniae* strain to make this possible. IR1 approached an identical strain of IR1 or other strains tested as rapidly as it did B12. This supports the conclusion that there is no discrimination between prey and non-prey bacteria before contact—i.e., there is no evidence for specific, directed gliding triggered by diffusible compounds from masses of prey bacteria attracting IR1. When IR1 encountered the motile strains *Flavobacterium* F52, *F. aquidurense* or *F. johnsoniae* there was no interpenetration of the two species (Fig. S6). This was also the case with the apparently non-motile *Flavobacterium* DD5b, suggesting that gliding motility is not involved in excluding IR1. IR1 neither invaded nor predated itself or other colonies of IR1, including non-motile, non-spreading IR1 mutants M17, M12 or M23 and spreading mutant M5. In addition, *F. johnsoniae* was predatory against B12 on low-salt plates (Table S3 and Fig. S6a) but did not infiltrate or predate other flavobacteria, including IR1, under these conditions (Fig. S6b, c).

### Mutants deficient in gliding motility are deficient in predation

**Fig. 5:**
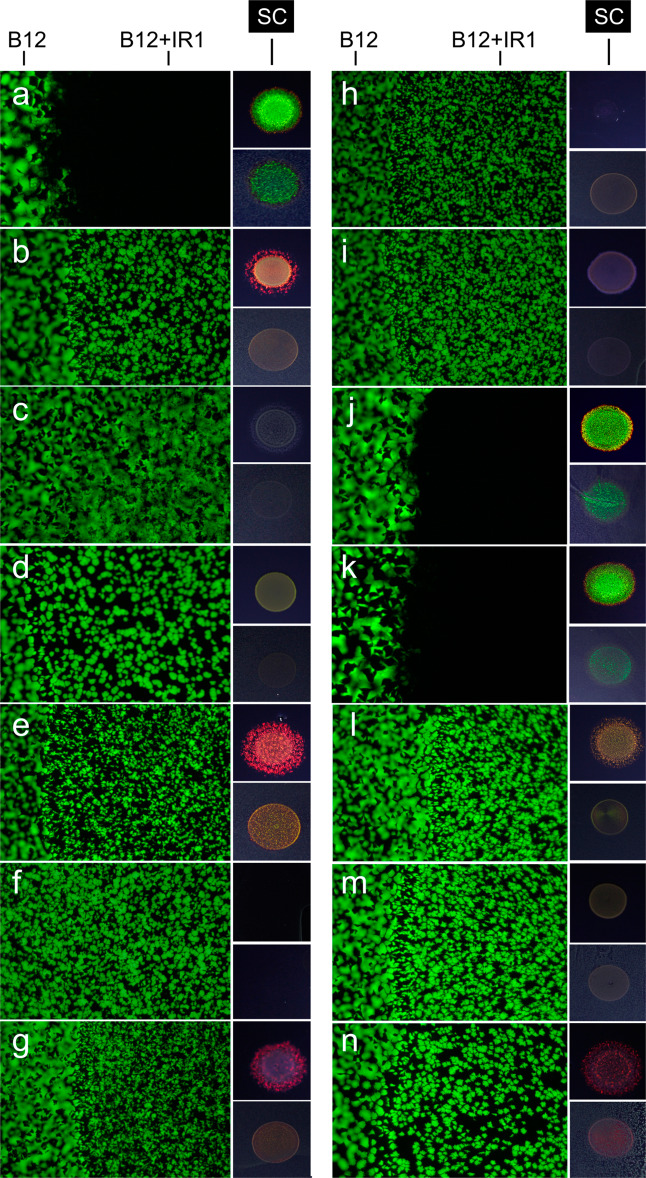
Competition by SC-deficient IR1 mutants. B12(pGFP) was mixed with IR1 (WT or transposon mutants) in the co-inoculation competition assay on ASWBLow agar. After 30 h the growth of B12 was assessed by fluorescence microscopy, capturing a series of 4 × 2.5 mm areas in triplicate (large images). Quantification of GFP was used to assess the degree to which IR1 inhibited the growth of B12 (Fig. S7). SC (structural colour) insets: two colony images for the same strain showing the appropriate strain cultured for 30 h on ASWBC agar (upper inset) or ASWBLow agar (lower inset). **a** WT IR1 strain. **b** M1 (*gmp1*::HiMar). **c** M5 (*spoT5*::HiMar)*. **d** M9 (*acr9*::HiMar). **e** M10 (*mal10*::HiMar). **f** M12 (*sprF12*::HiMar)*. **g** M16 (*hypA16*::HiMar)*. **h** M17 (*gldiA17*::HiMar)*. **i** M49 (*mtr49*::HiMar). **j** M51 (*malT51*::HiMar)*. **k** M52 (*hypB52*::HiMar)*. **l** M65 (*hk65*::HiMar). **m** M76 (*ugd76*::HiMar). **n** M160 (*hypC160*::HiMar). For strains marked * independent transposon insertions in the same gene were available and tested with similar results.

Transposon mutants of IR1 (Table S1) were tested for their ability to inhibit the growth of B12(pGFP) in co-inoculation assays. A HiMar transposon insertion into the gene encoding the SprF protein (M12, *sprF12*::HiMar) was sufficient to inhibit colony spreading and with no detectable gliding motility (as judged by confocal microscopy on agar). In addition, a transposon insertion in a novel gene cluster required for spreading and motility (M17, *gldiA17*::HiMar) gave a similar phenotype (Fig. 5 and Fig. S7). Both M12 and M17 were ‘dull’ mutants, i.e., showed reduced SC.

### SC mutants that retained motility failed to infiltrate or predate B12 in encounter assays

Two SC-deficient mutants that retained motility, M5 (*spoT5*::HiMar) and M16 (*hypA16*::HiMar), were tested in encounter assays against strain B12. Although M5 glides, the colonies are disorganized so that the cells no longer align effectively in a 2DPC and SC is largely eliminated [1]. M16 can be highly organized but with a different cell size and packing arrangement leading to a shift in SC on ASWBC agar [1], but under the test conditions on ASWLow plates SC was reduced. In both cases, no degradation of the B12 colony by these mutants was observed (Fig. S5b, c). In addition, microscopy and recovery of cells from the interior of the B12 colony followed by selective viable counts for IR1 both failed to demonstrate any penetration into the B12 colony. These results support the colocalization assays with the same mutants, that the ordering is required for predation.

### A broader role for cell organization in predation revealed by mutants of IR1

In addition to motility deficient mutants, mutants previously shown to be altered in SC were tested in colocalization assays. A wide range of mutants showing decreased SC on ASWBLow agar (without B12) were impaired in predation (Fig. 5 and Fig. S7a). In contrast, mutants with transposon insertions in genes that did not decrease the bright green SC typical of the WT competed as effectively as the WT. These data suggest a strong correlation between the ability to organize into a regular and densely packed mass of cells, i.e., a 2DPC perceived as an intense, angle-dependent green, and the ability to compete with other bacteria.

### Additional protein synthesis is required for SC-competent IR1 to predate B12

Co-inoculation assays were repeated under conditions in which protein synthesis of IR1 was blocked with antibiotics (clindamycin, tiamulin) which did not inhibit the growth of B12(pGFP). Under these conditions, IR1 still formed a 2DPC within 10–20 min, as effectively as in the absence of either antibiotic. However, despite a high density of IR1, the ability to predate B12 was largely lost (Fig. S8). These data suggest that cell organization is a prerequisite for efficient predation but the synthesis of additional proteins is also required, possibly triggered by encountering a suitable prey.

### Rare, spontaneous colonies in strain B12 are resistant to both IR1 and *F. johnsoniae* UW101, suggesting the involvement of a Type VI secretory system in killing

Spontaneous colonies of B12 were identified that were resistant to IR1 predation. These isolates were retested for predation by *F. johnsoniae* UW101, which has been previously shown to use a Type VI^iii^ secretory system to kill Gram-negative competitors [3]. Cross-resistance was observed (Fig. S7b), suggesting that IR1 was employing a similar killing mechanism to *F. johnsoniae*. Resistance was lost after passaging the isolates through liquid culture, suggesting a temporary phenotypic form of resistance to predation. BlastP searches of the IR1 genome suggested that core components of the Type VI^iii^ system from *F. johnsoniae* (*vgrG*, *clpV*, and the *tssB*, *C*, *E*, *F*, *G*, *K*, *N*, *O* and *P* genes as well as two copies of *hcp*) were also present in IR1.

### Predation of B12 by IR1 occurs on the surfaces of *Fucus vesiculosus*

A model cultivation system was set up using *F. vesiculosus* inoculated with a mixture of B12 (pGFP) and either WT or M5 IR1. After 30 h B12 was only found at the periphery of masses of WT IR1. In contrast, mixtures of M5 and B12(pGFP) were observed. This suggests that formation of a photonic crystal affects the segregation of IR1 and B12 on a naturally occurring surface which is consistent with the WT being a more efficient predator than the disorganized but motile mutant M5 (Fig. S9).

### Formation of a photonic crystal by IR1 does not protect it from predation by strain PIR4

**Fig. 6:**
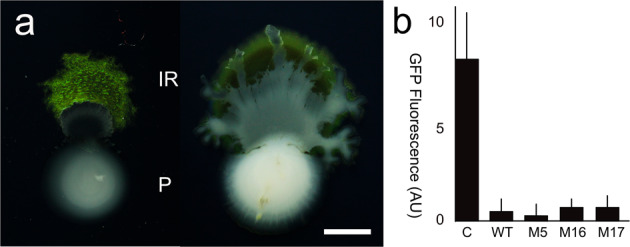
Predation of IR1 by *Rhodococcus spp*. PIR4. **a** Images of PIR4 (P, white) apparently moving towards and degrading a colony of IR1 (IR1 SC green) after 30 and 48 h (left and right, respectively). Scale bar indicates 5 mm. **b** Quantification of predation of GFP-expressing strains (WT and mutants) of IR1 by PIR4. C indicates a control (WT without PIR4). Replicates were threefold in arbitrary units of fluorescence; error bars indicate SD from the mean.

During the initial screening for bacteria interacting with IR1, an isolate, *Rhodococcus spp* PIR4 (PIR4) was obtained that was predatory on IR1 (Fig. 6). When adjacent to IR1, a colony of PIR4 invaded and degraded IR1 over a period of 48 h at 20 °C. This process appeared similar to IR1 predating B12 in the following respects: (i) the phenomenon occurred on low nutrient (ASWBLow and ASWBFLow) agar but not on richer nutrient medium (ASWBC plates) despite the rapid growth of both strains; (ii) placing a sterile PAO membrane between IR1 and PIR4 (similar to Fig. S4) completely inhibited the killing of IR1 by PIR4, suggesting a close ranged interaction and not production of a diffusible agent such as an antibiotic or toxin; (iii) on starvation medium the presence of IR1 increased the growth of PIR4 (up to fourfold to sixfold after 72 h determined by viable count), indicating PIR4 could grow at the expense of IR1. PIR4 was motile on ASWBLow agar; but this was only observed when >10^8^ cfu of IR1 cells were spotted within 5 mm, in which case motility appeared directed towards IR1 (Fig. 6). This suggests a degree of sensing and targeting of IR1; unlike the interaction of IR1 and B12, in which the initial collision between the strains appeared accidental, with the only specific interactions occurring after this event. Using a co-inoculation assay the ability of PIR4 to predate WT and mutant strains of IR1 (Fig. 6) was quantified. No significant differences were found, suggesting that motility and formation of a 2DPC did not provide resistance.

## Discussion

The estuarine and littoral environments have numerous surfaces upon which microorganisms spread and interact. Gliding flavobacteria have been implicated in predation of other bacteria, and microbe-on-microbe predation is of ecological importance [24–26, 28]. We investigated previously unknown predator–prey relationships of IR1 with other bacteria. IR1 is a broad-spectrum predator, attacking Gram-positive and Gram-negative bacteria and some yeasts. Unlike myxobacterial predation [17] there does not appear to be any sensory process mediating contact of IR1 with the prey colony, nor the process of engulfment. Surrounding of other colonies appears to be determined by physical interactions, with a tendency for IR1 to glide along the edges of colonies and other barriers. This still occurred in a mutant that was motile but both disorganized and dull (M5), suggesting no advantage to the formation of a 2DPC at this early stage in predation. Whilst simple, this mechanism of dispersal and engulfment is effective. Moving along an edge can increase the dispersal rate up to fivefold and completely surround and restrict and degrade competing microorganisms. Microbial surface motility is generally studied on a flat agar surface. In contrast, any plausible surface environment of IR1 (including macroalgae) is likely to have significant topography. A textured environment is normally considered a problem for microbial dispersal [29]. However, we suggest that some “rough terrain” may provide “rapid transit highways” for gliding bacteria. After contact, IR1 infiltrated the colonies of prey bacteria. Once significant numbers of cells penetrated the target colony, IR1 proceeded by undercutting the potential prey microorganisms, separating the latter from the source of nutrients which may also contribute to a successful competitive strategy. Undercutting has not to our knowledge been previously described and may be an important method of competing on nutrient-rich surfaces, such as macroalgae.

SC, resulting from light interacting with organized nanostructures, is common within the flavobacteria [1, 5, 6, 27]. The process of organization requires energy [1, 5, 6] and considerable gene investment [1]. However, to date no competitive advantage of bacterial SC has been demonstrated. In this work we have shown that formation of a 2DPC, in which cells approach their maximum packing density, is a prerequisite for efficient predation of other bacteria. However, SC was not sufficient for predation in itself; after contact between IR1 and prey other proteins seem to be induced. It is possible that the undercutting and killing of the prey colony may be most effectively performed by gliding bacteria in close ranks. This was in contrast to the converse situation: when IR1 was the target of predation by PIR4, the dense, highly aligned organization of cells did not confer any protection. Rhodococci are not normally considered motile. This work indicates that a cryptic surface motility or translocation mechanism is triggered in PIR4 by nearby colonies of IR1. This suggests a previously unsuspected ecological role for rhodococci.

Whilst indirect, this is the first evidence for a biological function for SC in bacteria. The majority of eukaryotic SCs are involved in communication, for example the sexual selection of the peacock by colourful feathers or other visual properties such as camouflage. However, Eukaryotic photonic nanostructures can also confer lubrication, hydrophobicity and photoprotection, with the associated SC apparently coincidental [10]. IR1 may add predation to this list of colourful accidents, but we do not exclude properties of SC directly related to illumination in these highly organized and dynamic colonies of iridescent flavobacteria.

## Materials and methods

### Culture conditions

Growth of all flavobacterial strains was at 20 °C under aerobic conditions unless stated otherwise. ASWBLow was used as the basal medium. Alternatively, ASWB, ASWBC, and ASWVLow media were used as noted and as previously described [1]. Starvation medium contained (per litre) 10 g KCl (Sigma-Aldrich), 15 g agar (Invitrogen) and 10 mg yeast extract (Sigma-Aldrich). Culture under illuminated conditions used a 50 W broad-spectrum white LED lamp at 20 cm distance using a water filter to prevent heating. Cultures in the dark were enclosed in aluminium foil.

### Strain identification

Isolates were identified by sequencing after PCR amplification of the variable regions of DNA encoding 16S rRNA [30, 31]. The sequences were comparatively analyzed using the BLAST function of GenBank, RDP (Ribosomal Database Project) release 11 [32] and SILVA rRNA database project [33].

### Competition experiments 1: liquid culture

Bacteria (*Flavobacterium* IR1 WT or mutants*, F. johnsoniae* UW101, *E. cloacae* B12) were cultured in broths with different amounts of nutrients: ASW broth (rich), ASWLow broth (intermediate) and ASWVLow (low nutrients). Inoculations of 5 × 10^5^ cfu of pairs of strains (generally B12 vs a *Flavobacterium*) were in 20 ml of broth incubated at 20 °C for 24 h, with selective viable counts used to determine the results of competition.

### Competition experiments 2: biofilm

For biofilm assays, 5 × 10^5^ cfu of each strain were incubated in six-well polycarbonate plates without shaking at 20 °C. Culture media (4 ml of rich, intermediate, low) were as described above. After 72 h, the culture medium was removed and the plates washed once with sterile 1% (w/v) KCl. The cells adhering to the wells were then resuspended in sterile KCl and subjected to selective viable counting.

### Competition experiments 3: outgrowth on agar

A total of 5 × 10^5^ cells of IR1 and B12 were co-inoculated as a 5-mm-diameter spot on 1.5% (w/v) agar with nutrients. Recovery of cells from within the original inoculation area was made with a sterile loop for subsequent selective viable counting. In addition, cells were recovered from outside the region of inoculation to assess representation of the two strains during outgrowth and spreading.

### Competition experiments 4: co-inoculation on agar

B12(pGFP) was inoculated on agar as a large spot or covering the entire plate with 5 × 10^5^ cells cm^−2^. After drying, 5 × 10^5^ cells of IR1 were inoculated in a 4-µl, 5-mm-diameter spot. Imaging of GFP by fluorescence microscopy was used to acquire data, with image analysis by ImageJ.

### Competition experiments 5: co-inoculation on agar

These were performed and analyzed essentially as the previous paragraph but with spots of PIR4 (10^6^ cells) on a lawn of IR1-expressing GFP (2 × 10^6^ cells cm^−2^) to assess predation of IR1 by PIR4.

### Competition experiments 6: co-inoculation on *Fucus vesiculosus*

Culture on the surface of macroalgae, cut into 5 × 5 mm pieces, was as previously described [1]. Co-inoculation of mixtures of IR1 and B12(pGFP) was by applying mixtures of the two strains (5 × 10^5^ cells of each in a 5 µl spot) with incubation as 20 °C and imaging after 30 h.

### Competition experiments 7: predation (encounter) assays

Predation assays were performed by spotting IR1 (6 × 10^7^ cfu) in a 0.5-cm-diameter spot on an ASWLow agar plate (1.5% w/v agar) 3 mm away from a similar spot of B12(pGFP) or other prey bacterium and incubating for up to 24 h. Selective viable counts and/or imaging by fluorescence or confocal microscopy was used to determine outcomes. To localize killing of B12(pGFP) by IR1, PI (Life Technologies) was used to selectively stain damaged cells [34, 35]. At the appropriate time point, a 4-µl spot of 0.1 mM PI was placed 2 mm away from the region to be imaged and allowed to diffuse through the agar. After this time, the region of interest was imaged by fluorescence or confocal microscopy. In addition, recovery of cells and imaging of dilutions on microscope slides were done to assess the staining of the two strains. Similar assays were performed using adjacent spots of PIR4 and IR1 to assess predation of the latter by the former.

### Assay for the role of a diffusible toxin in predation

Agar plates containing a barrier constructed of a porous ceramic were used to test whether a diffusible compound mediated predation of B12 by IR1 [35] (Fig. S4). This set up separated IR1 from B12 by a 60 µm thick wafer of porous aluminium oxide which could be penetrated by small molecules but not bacteria [30], with antibiotic-containing tabs used to prevent this barrier being circumvented (Fig. S4). IR1 was inoculated one side of the barrier and B12 the other as described under encounter assays and the effect of this barrier on B12 assessed by fluorescence microscopy.

### Selective viable counts

The proportion of IR1 and B12 in competition experiments was determined by viable counts under conditions selective to one of the two species. *E. cloacae* B12 was selected for by growth at 37 °C on ASWBLow agar. Flavobacteria were selected on ASWBLow plates containing 10 µg ml^−1^ ampicillin, 10 µg ml^−1^ colistin and 2 mM sucrose for 2 days at 20 °C.

### Microscopy and image processing

Images were captured with an Olympus BX-41 microscope and quantification was by ImageJ (version 1.52) as previously described [30, 36]. For confocal microscopy imaging, 6 × 6 mm agar pieces were inverted onto an imaging chamber (MatTek, P35G-1.5-14-C). Confocal fluorescence images were acquired at room temperature on a Nikon A1R inverted confocal laser scanning microscope equipped with a Nikon SR Apo TIRF 100×/1.49 NA oil immersion objective lens. GFP and PI fluorescence were excited using respectively the 488 nm and the 561 nm laser lines and were detected with two separate GaAsP detectors and emission filters, 525/50 nm and 595/50 nm, respectively. In the case of mixed populations of bacteria stained/non-stained, the scanned excitation laser light was collected in transmission using the widefield condenser of the microscope stand and detected with an additional photo-multiplier tube to get a morphology contrast of non-stained bacteria.

### Assays for the involvement of colony edges and capillarity

Simulated colonies were created from drops of candle wax (Sigma, NL). Microscope slides sterilized with ethanol were used to create edges for IR1 when placed on agar plates. Slides were siliconized (Sigmacote) according to the manufacturer’s instructions (Sigma-Aldrich) to render them hydrophobic.

### Predation-resistant strain selection in B12

Resistant colonies of B12 were selected for resistance to IR1 predation by spreading mixtures of the two strains on ASWLow plates (10^8^ cfu B12(pGFP) and 10^9^ cfu IR1) and incubating for 4 days at 20 °C. Resistant colonies of B12 were retested for resistance to IR1 and *F. johnsoniae* UW101 in co-inoculation assays.

## Supplementary information

ISME Supplemental Figs

ISME Supplemental Tables PLUS

SI Video 1

SI Video 2
