# Supplementary material for: The cell organization underlying structural colour is involved in Flavobacterium IR1 predation
Source: ISME J. 2020 Sep 1;14(11):2890–900. doi: 10.1038/s41396-020-00760-6 (PMC7784876; doi:10.1038/s41396-020-00760-6)
Supplement: Supplementary file 1 — ISME Supplemental Figs [file 41396_2020_760_MOESM1_ESM.docx]

**­­SUPPLEMENTARY FIGURES**

**
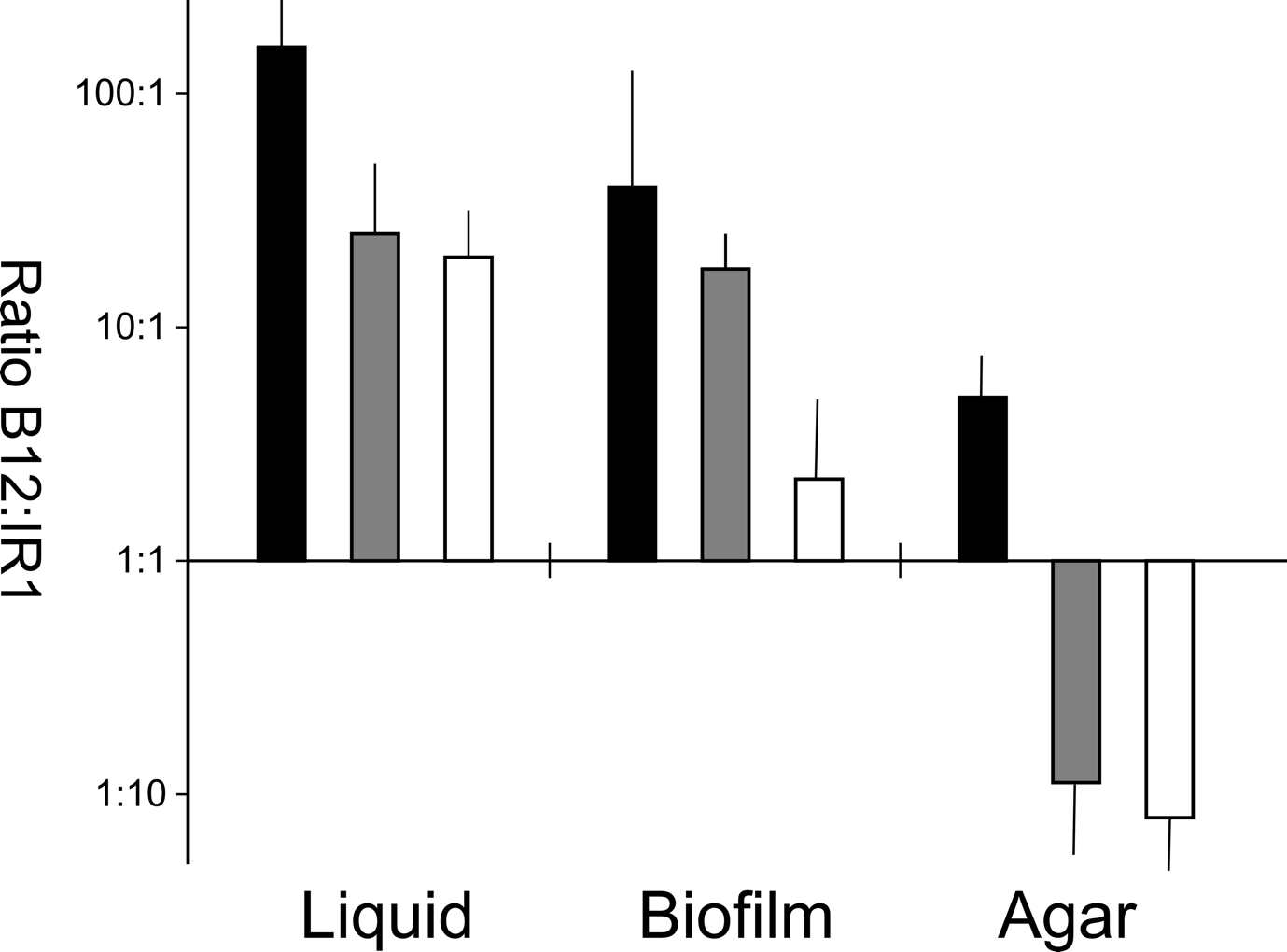
**

**FIG S1** Competition between IR1 and B12 under different growth conditions. The final ratios between the two co-inoculated strains (1:1) were calculated by selective viable counts (n=3, +/- SD from the mean) after 48 h at 20 °C in shaken liquid culture, in a biofilm model or on agar plates (with 1.5% w/v agar). Black bars; rich medium (ASWBC). Grey; low nutrient medium (ASWBLow). White bars; very low nutrient medium (ASWBVLow).


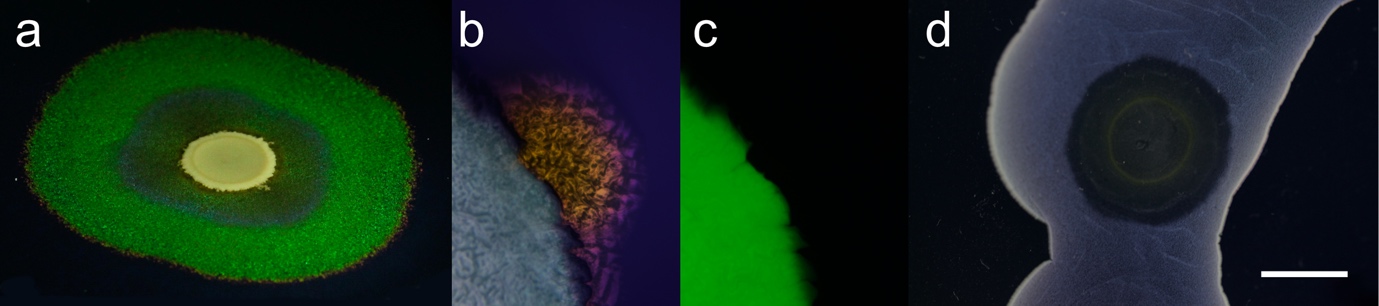


**FIG S2.** Examples of competition between IR1 and B12. (a) Co-inoculation of IR1 and B12(pGFP) on ASWLow plates (2% w/v agar) using a ratio of 1:100. Despite IR1 being in the minority the culture moving away from the inoculation spot is composed of pure IR1 after 3 days. (b) Image from the edge of the colony from panel (a) after 26 h showing the outgrowth of IR1 (orange-pink structural colour). (c) As (b) but imaging B12 by fluorescence microscopy. (d) Example of scavenging. IR1 was inoculated on a lawn of heat-killed B12 on starvation medium and incubated for 9 days. The result is spreading of the IR1 colony which only occurs on this medium when B12 cells are available as a nutrient source. Scale bar in panel (d) indicates 0.5 cm for (a), 0.5 mm for (b) and (c) and 1.6 cm for (d).


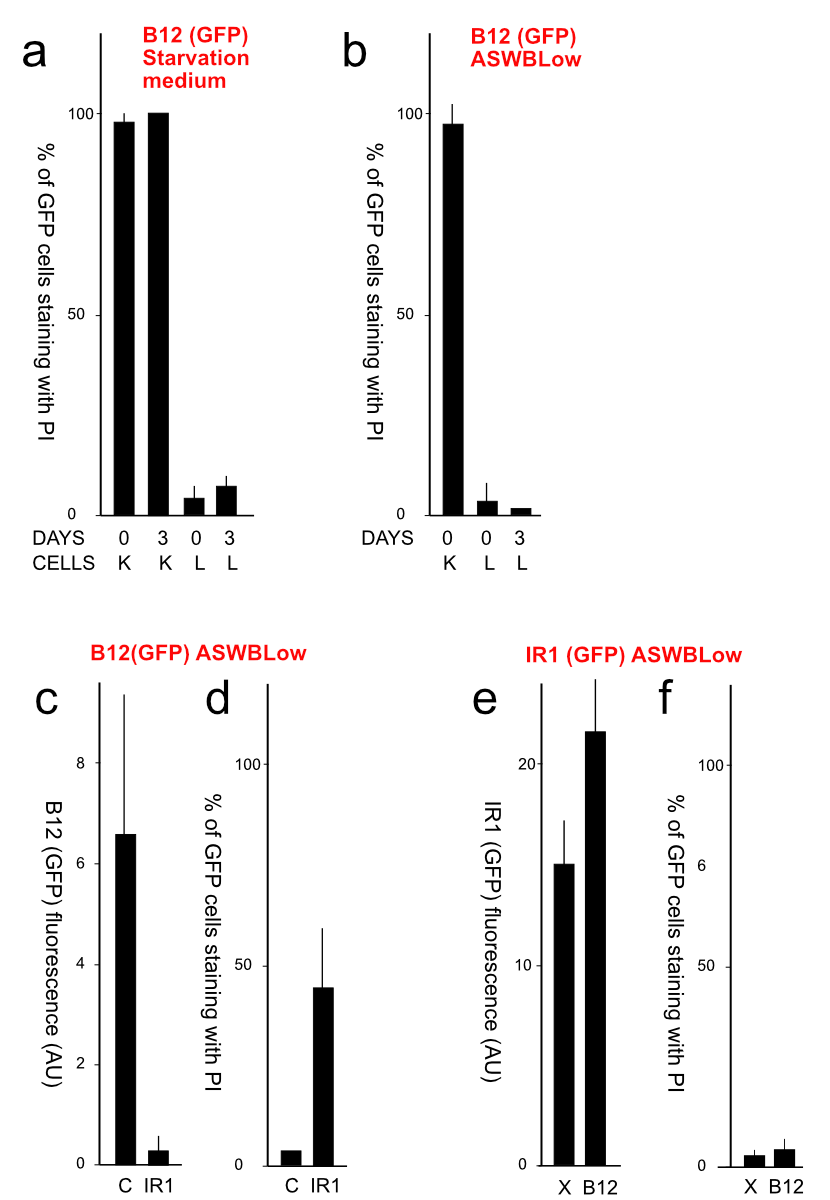


**FIG S3.** Experiments on viability of strains during predation assays. (a) Propidium iodide (PI) staining of cells sampled from strain B12 incubated on starvation medium in the absence of IR1 and imaged by fluorescence microscopy. Incubation times are in days. CELLS, K = control of killed cells and L = inoculum of living cells. (b) PI staining of B12 inoculated on ASWLow agar and sampled, stained and images after 0 or 3 days. K = cells killed before inoculation. (c) Coinoculation experiment in which strain B12(pGFP) is spread on ASWLow agar and spotted with WT1 IR1 with the overall level of B12 fluorescence measured from microscopy images after 30h (Fig. 5). C = control without IR1, IR1 = with IR1. (d) PI staining of cells from the co-inoculation region in the experiment from the previous panel. C = control without IR1, IR1 = with IR1. (e) Experiment similar to panel c but spotting a GFP expressing strain of IR1 on a lawn of B12 without GFP. X = IR1 inoculated on a plate without B12. B12, with lawn of this strain. (f) Experiment similar to d but inverted, i.e. using GFP expressing IR1 and B12 without GFP. X = IR1 inoculated on a plate without B12. Average of three replicates +/- S.D for all panels.

**
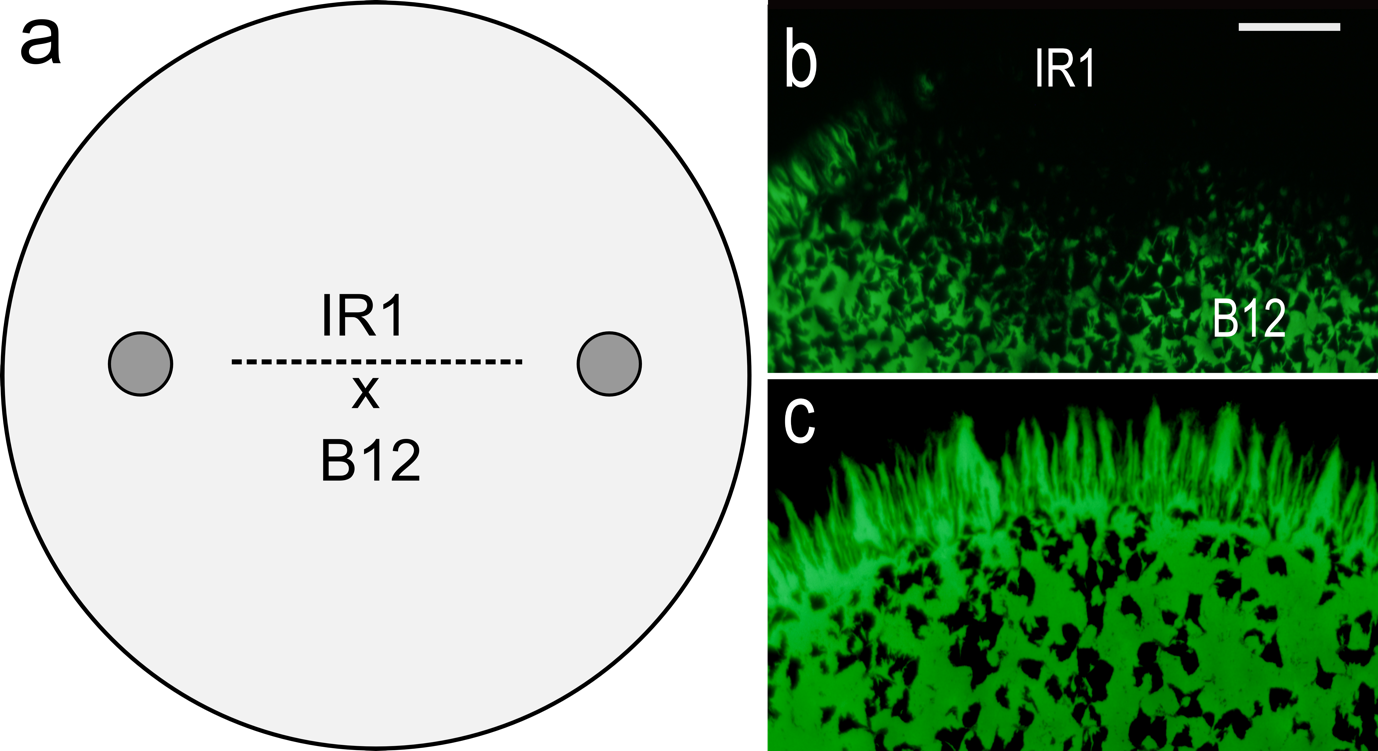
**

**FIG S4.** Predation by IR1 against B12 is close range. (a) Set-up for separation of B12 and IR1 by a porous ceramic membrane showing an agar plate viewed from above. IR1 and B12(pGFP) inoculation sites are shown with the imaging point marked with an x (1.5 mm from the membrane on the B12 side). A porous aluminium oxide membrane (dashed horizontal line) was positioned to create a 36 mm long barrier flanked with tetracycline-containing Neosensitabs (grey shaded circles) positioned to prevent IR1 gliding around the PAO membrane. (b) Fluorescence image of B12(pGFP) at position x from a control experiment lacking the intervening PAO membrane. (c) Fluorescence image of B12(pGFP) at position x with the intervening PAO membrane, showing no sign of inhibition of B12 after 4 days. Scale bar in panel b indicates 0.7 mm for panels b and c.


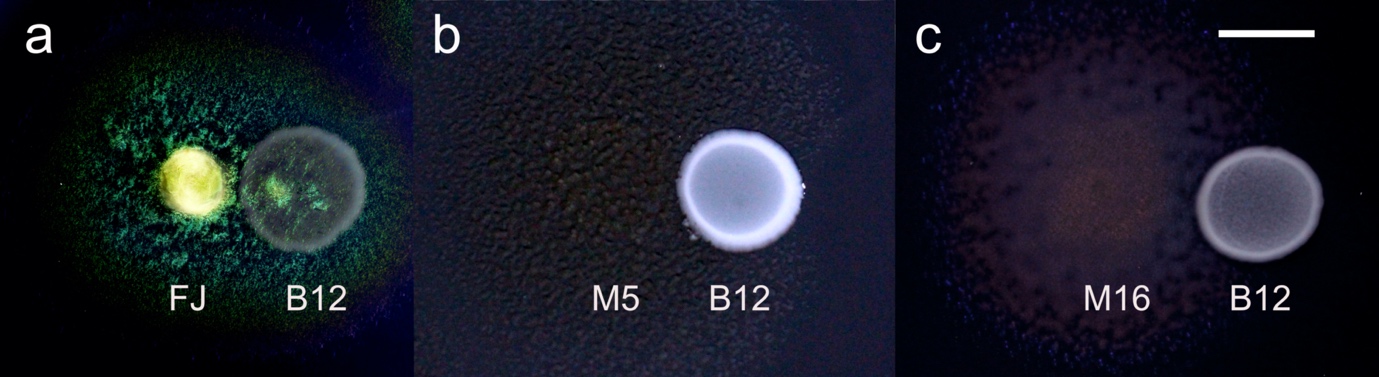


**FIG S5** (a) Invasion of strain B12 by *F. johnsoniae* UW101 (FJ) on ASWBLow agar without KCl. *F. johnsoniae* UW101 shows structural colour on this medium (orange/yellow towards the colony centre and then green with a halo of pink/purple). (b) Encounter assay between IR1 mutant M5 on ASWBLow agar which shows reduced SC and no invasion or degradation of the B12 colony. (c) As panel b but IR1 mutant M16 replacing M5. Scale bar in panel c indicates 5 mm for all panels. Because the mutant strains show poor visibility compared to SC strains, additional checks were made to test whether mutants M5 or M16 penetrated the B12 colony. Recovery of cells from the centre of the B12 colony and culture on selective medium did not result in colonies of M5 or M16 suggesting this was not occurring. See Fig 2a in the main text for a comparable experiment from WT IR1.


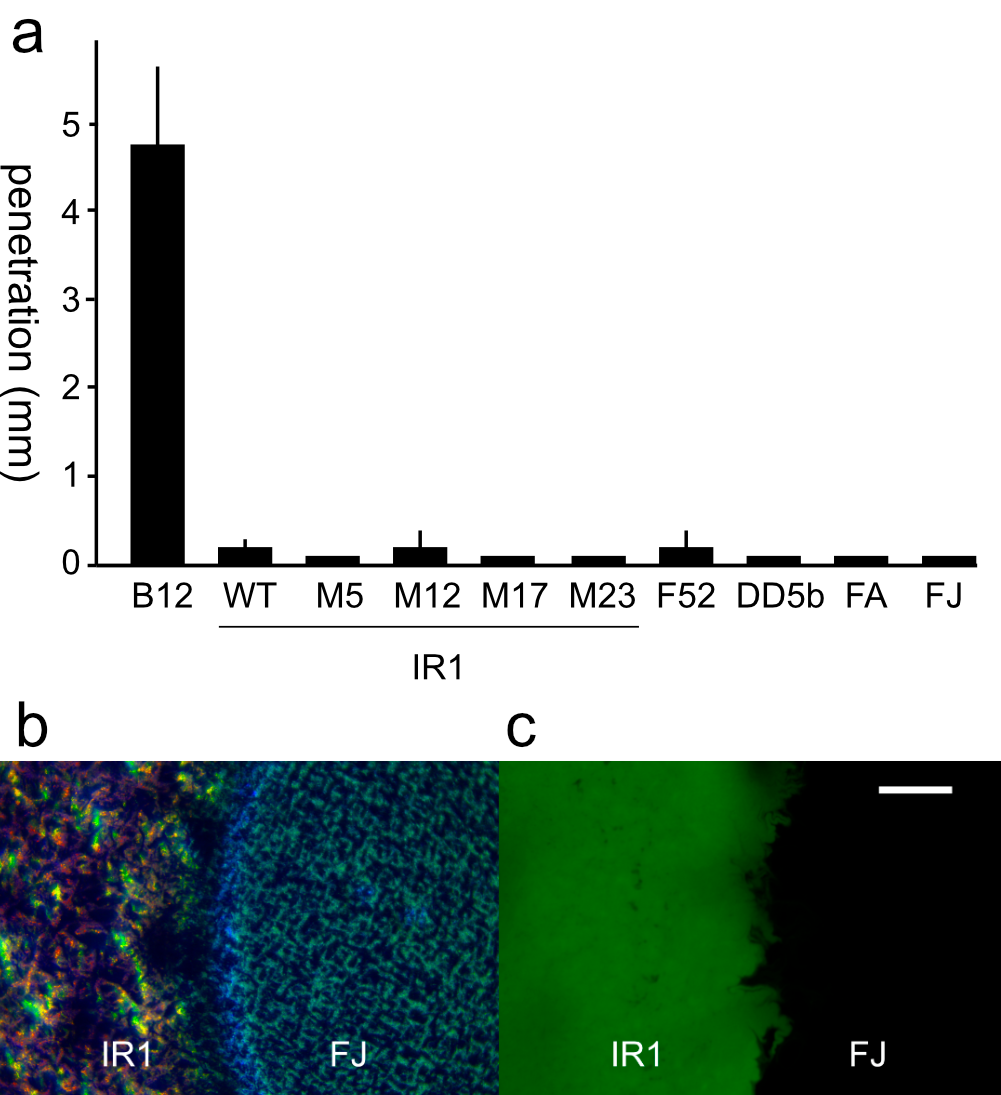


**FIG S6.** (a) Invasion of flavobacterial strains and B12 by IR1. Aliquots (4 µl) of the two strains (IR1 expressing GFP) on an ASWBLow agar plate, with the neared colony edges 3 mm apart, and incubated for 3 days at 22 °C. The border between the two strains was imaged by microscopy for GFP and structural colour. The greatest distance of penetration from the periphery of the target colony was calculated from TIFF Images using Image J to calculate distances as the average of three measurements +/- SD**.** WT, wild type IR1; M5-M23, IR1 mutants; F52, *Flavobacterium* F52; DD5b, *Flavobacterium succinans*; FA. *Flavobacterium aquidurense*; FJ, *Flavobacterium johnsoniae* UW1010*~~.~~* Average of three replicates +/- S.D. (b) and (c) Example of the experiment described in panel a showing limited interpenetration of *F. johnsoniae* and IR1. (b) IR1 and *F. johnsoniae* UW101 (FJ) meeting on ASWLow agar without KCl, under which conditions both strains are predatory and display distinctive structural colour but fail to interpenetrate. (c) As (b) but with IR1 expressing GFP, imaged by fluorescence microscopy. Scale bar for panels (b) and (c) (upper right of c) represents 0.1 mm.

**
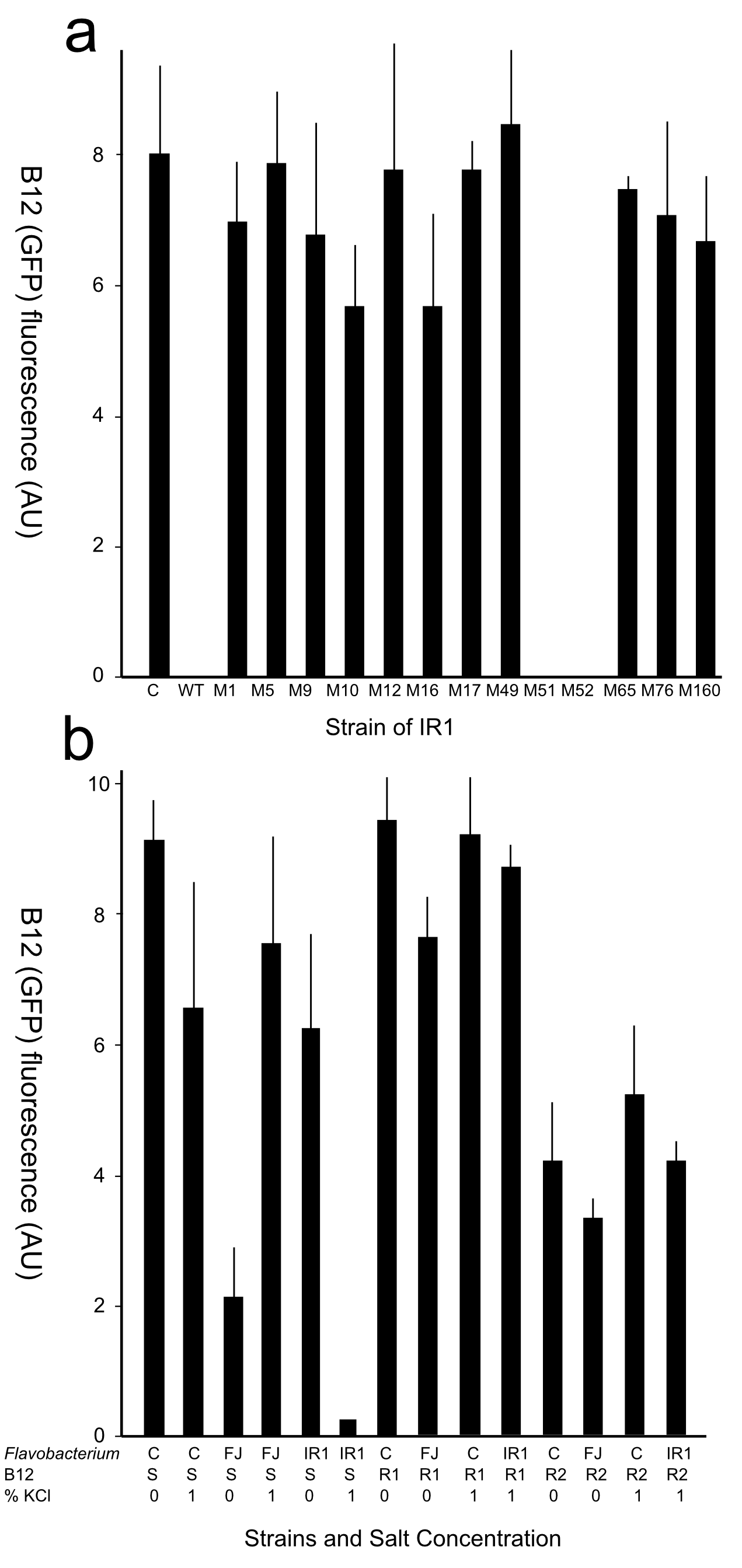
**

**FIG S7.** Predation assays by co-inoculation method quantified using GFP expression of B12. (a) Competition by mutants of IR1 related to structural colour. B12(pGFP) was mixed with IR1 (WT) or transposon mutants of IR1 in the standard competition assay on ASWBLow agar. After 30 h the growth of B12 was assessed by fluorescence microscopy. These data represent a quantification of the fluorescence images (with additional replicates) shown in Figure 5 of the main text. (b) Predation of B12 by *F. johnsoniae* UW101 and IR1 on B12(pGFP) sensitive and resistant lineages. Flavobacteria present are C (Control, no Flavobacteria), FJ (*F. johnsoniae* UW101) and IR1. B12(pGFP) is S (predator sensitive lineage, as used generally throughout this work) and R1 or R2 (resistant, two colonies selected on the basis of surviving predation by IR1). % KCl (w/v) in the agar test plates is as noted. Average of three replicates +/- S.D for both panels.

**
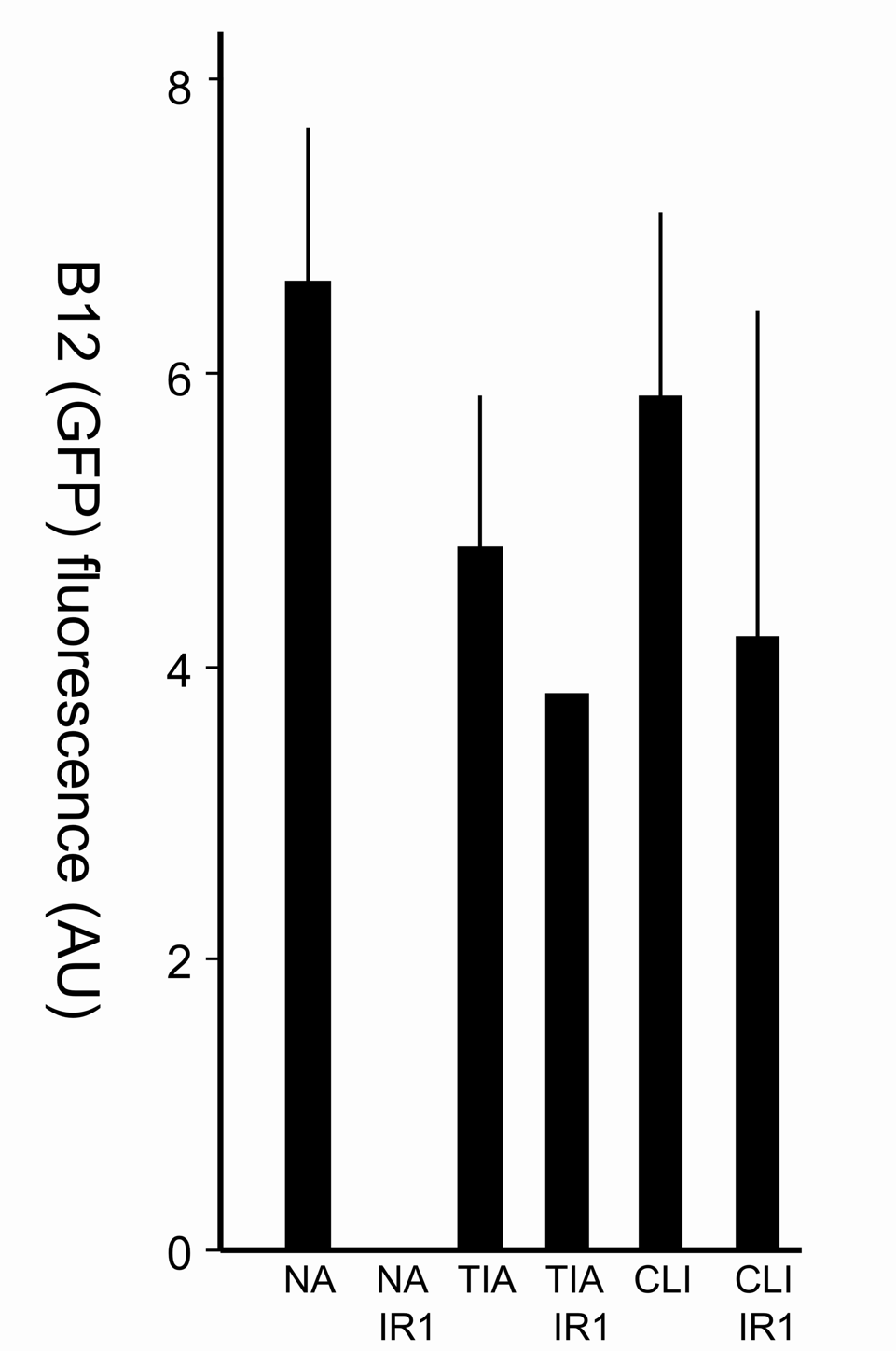
**

**FIG S8.** Predation assays by co-inoculation method quantified using GFP expression. Predation (but not re-formation of structural colour from a mixed/disorganised colony) is blocked by inhibiting protein synthesis in structural colour-competent cells of IR1. These assays were performed on plates containing antibiotics which block protein synthesis and growth of IR1 but do not inhibit the capacity of IR1 to form structural colour nor the growth of B12. IR1 present where noted. NA, no antibiotics; CLI, 1 µg ml^-1^ clindamycin, TIA, 1 µg ml^-1^ tiamulin. Average of three replicates +/- S.D.


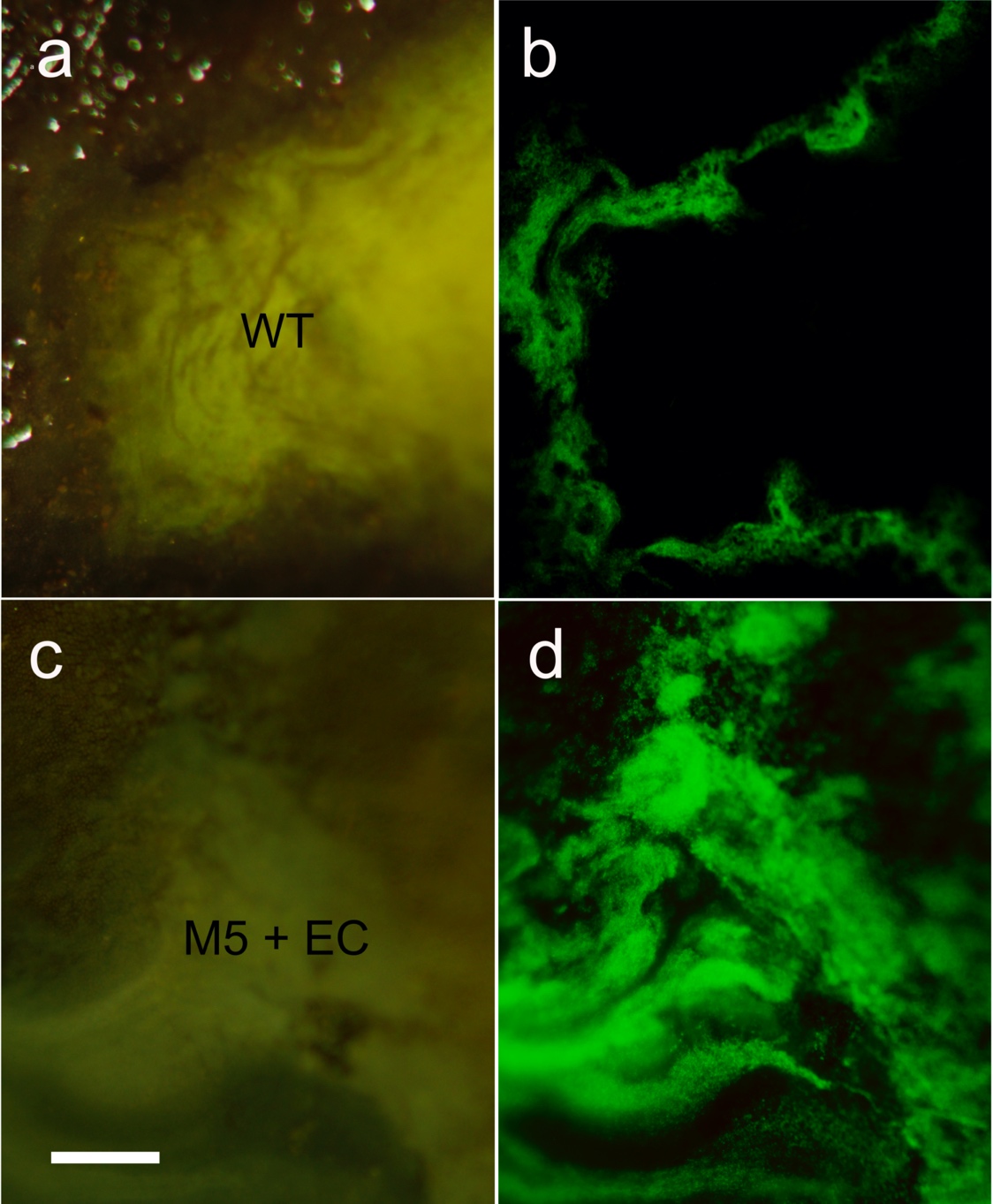


**FIG S9.** Visualization of IR1 and B12 co-inoculated on the surface of the brown algae *F. vesiculosus*. (a and b) WT IR1 combined with B12 with imaging after 30 h by epi-illumination. (a) Visualization of IR1 (WT) using white light from the side and (b) fluorescence microscopy for B12. (c and d) As the previous experiment but with IR1 M5 with *Enterobacter cloacae* B12 (M5+EC) The disorganised, duller M5 largely colocalizes with B12 whilst the WT segregates. Scale bar in panel c indicates 0.5 mm for all panels.

**MOVIE S1.** Confocal imaging of B12 (expressing GFP) and IR1 (unlabelled) interacting at low cell density over 2 min. Cells of IR1 can be seen moving between even quite closely spaced B12 by gliding motility. Scale bar indicates 20 µm.

**MOVIE S2.** Confocal imaging of B12 (expressing GFP) and IR1 (unlabelled) interacting at a high cell density over 2 min during the process of IR1 invading a colony of B12 (e.g. in direction of arrow). Scale bar indicates 20 µm.
