# Supplementary material for: The cell organization underlying structural colour is involved in Flavobacterium IR1 predation
Source: ISME J. 2020 Sep 1;14(11):2890–900. doi: 10.1038/s41396-020-00760-6 (PMC7784876; doi:10.1038/s41396-020-00760-6)
Supplement: Supplementary file 2 — ISME Supplemental Tables PLUS [file 41396_2020_760_MOESM2_ESM.docx]

**Strain Relevant Genotype Notes Source/Reference**

*Flavobacterium* IR1 WT Bright green structural colour (SC), gliding (1)

*Flavobacterium* IR1 M1  *gmp1*::HiMar Reduced SC (dull), red shift (1)

*Flavobacterium* IR1 M5 *spoT5*::HiMar, Erm^R^  SC KO, motile (1)

*Flavobacterium* IR1 M9  *acr9*::HiMar Reduced SC (dull) (1)

*Flavobacterium* IR1 M10  *mal10*::HiMar Red shifted SC, motile (1)

*Flavobacterium* IR1 M12 *sprF12*::HiMar, Erm^R^  SC dull, non-spreading, motility deficient (1)

*Flavobacterium* IR1 M16 *hypA16*::HiMar, Erm^R^  SC red shift, motile (1)

*Flavobacterium* IR1 M17 *gldiA17*::HiMar, Erm^R^  SC dull, motility deficient (1)

*Flavobacterium* IR1 M23 *sprB*::HiMar, Erm^R^  SC KO, motility deficient (1)

*Flavobacterium* IR1 M47 *hypX47*::HiMar, ErmR Dull, motile, transposon insertion in NRPS^1^ (1)

*Flavobacterium* IR1 M49 *mtr49*::HiMar SC KO (1)

*Flavobacterium* IR1 M51 *malT51*::HiMar SC and gliding as WT (1)

­*Flavobacterium* IR1 M52 *hypB52*::HiMar SC and gliding as WT (1)

*Flavobacterium* IR1 M65 *hk65*::HiMar Dull, red, motile (1)

*Flavobacterium* IR1 M76 *ugd76*::HiMar Dull (1)

*Flavobacterium* IR1 M160 *hypC160*::HiMar Motile, sparse red SC (1)

*F. johnsoniae* UW101 (2)

*F. aquidurense* DSM 18293 (3)

*Flavobacterium* F52 (4)

*Flavobacterium succinicans* DD5b (5)

*Enterobacter cloacae* B12 Environmental isolate This study

*E. cloacae* B12 (pGFP) Amp^R^ Constitutively expresses GFP from plasmid This study

*Escherichia coli* X2 Amp^R^ Source of *gfpmut*2 used in pGFP and pLC001 (6)

*E. coli* (pLC001) ­ Amp^R^ (Tet^R^ in IR1) Constitutive GFP in IR1 and *E. coli* shuttle

vector based around pCP23 This study and (7)

*Flavobacterium* IR1 (GFP) Tet^R^ WT IR1 expressing GFP from plamid pLC001 This study

*Flavobacterium* IR1 M5 (GFP) Tet^R^, Erm^R^  Transposon mutant M5 expressing GFP This study

*Flavobacterium* IR1 M16 (GFP) Tet^R^, Erm^R^ Transposon mutant M16 expressing GFP This study

*Flavobacterium* IR1 M17 (GFP) Tet^R^, Erm^R^ Transposon mutant M17 expressing GFP This study

**TABLE S1.** Flavobacteria and genetically engineered strains used in this study.

**Strain Description ^1^Invasion ^2^Predation Source**

*Enterobacter cloacae* B12 Gram-negative bacterium + + This study

*Moraxella osloensis* Gram-negative bacterium + + This study

*Staphylococcus pasteuri* Gram-positive bacterium + + This study

*Proteus mirabilis* 3R Gram-negative swarming bacterium + - (8)

*Enterobacter aerogenes* GA2 Gram-negative bacterium + + (9)

*Escherichia coli* V001 Gram-negative bacterium + + (9)

*Klebsiella pneumoniae* B1 Gram-negative capsulated bacterium + - (9)

*Candida albicans* 4208 Yeast + + (10)

*Candida glabrata* 1925 Yeast + - (8)

*Rhodobacter sphaeroides* DSM158 Gram-negative photosynthetic bacterium + + DSMZ (DE)

*Paenibacillus vortex* Gram-positive swarming bacterium + + (10)

*Fusarium oxysporum*

f.sp. *tulipae* 5B8 CBS 118729  Filamentous fungus + - Westerdijk Centre (NL)

*Flavobacterium johnsoniae* UW101 Gram-negative gliding Flavobacterium - - Mark McBride (USA)

*Flavobacterium* F52 Gram-negative gliding Flavobacterium - - (4)

*Flavobacterium aquidurense* DSM 18293 Gram-negative gliding Flavobacterium - - DSMZ (DE)

*Flavobacterium succinans* DD5b Non-motile Flavobacterium - - (5)

*Rhodococcus spp.* PIR4 Bacterium predatory on IR1 NA - This study

**TABLE S2.** Summary of strains tested for invasion and predation by IR1.

^1^Invasion and ^2^predation columns indicate whether colonies of this strain are invaded or killed by IR1.

**Strain Description Invasion Predation Invasion Predation**

**% (w/v) KCl: 0 0 1 1**

*Candida albicans* 3007 Yeast + + - -

*Paenibacillus vortex* Gram-positive swarming bacterium + + - -

*Flavobacterium* IR1 Gram-negative gliding Flavobacterium - - - -

*Enterobacter cloacae* B12 Gram-negative bacterium + + - -

**Table S3: Predation by *F. johnsoniae* UW101**

Invasion and predation columns indicate whether colonies of this strain are invaded or killed by *F. johnsoniae* tested on plates with and without KCl.

**Supplementary References**

1. Johansen VE, Catón L, Hamidjaja R, Oosterink E, Wilts BD, Rasmussen TS, Sherlock MM, Ingham CJ, Vignolini S. 2018. Genetic manipulation of structural color in bacterial colonies. Proc Natl Acad Sci U S A 115:2652-2657.

2. McBride MJ, Xie G, Martens EC, Lapidus A, Henrissat B, Rhodes RG, Staroscik AM. 2009. Novel features of the polysaccharide-digesting gliding bacterium *Flavobacterium johnsoniae* as revealed by genome sequence analysis. Appl Environ Microbiol 75:6864-6875.

3. Cousin S, Paeuker O, Stackebrandt E. 2007. *Flavobacterium aquidurense* sp. nov. and *Flavobacterium hercynium* sp. nov., from a hard-water creek. Int J Syst Evol Microbiol 57:243-249.

4. Kolton M, Green SJ, Harel YM, Sela N, Elad Y, Cytryn E. 2012. Draft genome sequence of *Flavobacterium* sp. strain F52, isolated from the rhizosphere of bell pepper (*Capsicum annuum* L. cv. Maccabi). J Bacteriol 194:5462-5463.

5. Poehlein A, Najdenski H, Simeonova DD. 2017. Draft genome sequence of *Flavobacterium succinicans* Strain DD5b. Genome Announc 5:e01492-16.

6. Yagur-Kroll S, Schreuder E, Ingham CJ, Heideman R, Rosen R, Belkin S. 2015. A miniature porous aluminum oxide-based flow-cell for online water quality monitoring using bacterial sensor cells. Biosens Bioelectron 64:625-632.

7. Agarwal, S., Hunnicutt, D.W. and McBride, M.J., 1997. Cloning and characterization of the Flavobacterium johnsoniae (Cytophaga johnsonae) gliding motility gene, gldA. *Proceedings of the National Academy of Sciences*, *94*(22), pp.12139-12144.

8. Budding AE, Ingham CJ, Bitter W, Vandenbroucke-Grauls CM, Schneeberger PM. 2009. The Dienes phenomenon: competition and territoriality in swarming *Proteus mirabilis*. *J Bacteriol* 191:3892-3900.

9. Finkelshtein A, Roth D, Jacob EB, Ingham CJ. 2015. Bacterial swarms recruit cargo bacteria to pave the way in toxic environments. MBio 6(3):e00074-15.

10. Ingham CJ, Jacob EB. 2008. Swarming and complex pattern formation in *Paenibacillus vortex* studied by imaging and tracking cells. BMC Microbiol 8:36.
